# Susceptibility of *Drosophila suzukii* larvae to the combined administration of the entomopathogens *Bacillus thuringiensis* and *Steinernema carpocapsae*

**DOI:** 10.1038/s41598-021-87469-4

**Published:** 2021-04-14

**Authors:** Maristella Mastore, Silvia Quadroni, Maurizio F. Brivio

**Affiliations:** 1 Laboratory of Comparative Immunology and Parasitology, Department of Theoretical and Applied Sciences, University of Insubria, Varese, Italy; 2 Laboratory of Ecology, Department of Theoretical and Applied Sciences, University of Insubria, Varese, Italy

**Keywords:** Invasive species, Entomology

## Abstract

Non-native pests are often responsible for serious crop damage. Since *Drosophila suzukii* has invaded North America and Europe, the global production of soft, thin-skinned fruits has suffered severe losses. The control of this dipteran by pesticides, although commonly used, is not recommended because of the negative impact on the environment and human health. A possible alternative is the use of bio-insecticides, including *Bacillus thuringiensis* and entomopathogenic nematodes, such as *Steinernema carpocapsae*. These biological control agents have a fair effectiveness when used individually on *D. suzukii*, but both have limits related to different environmental, methodological, and physiological factors. In this work, we tested various concentrations of *B. thuringiensis* and *S. carpocapsae* to evaluate their efficacy on *D. suzukii* larvae, when administered individually or in combination by using agar traps. In the combined trials, we added the nematodes after 16 h or concurrently to the bacteria, and assessed larvae lethality from 16 to 48 h. The assays demonstrated a higher efficacy of the combined administration, both time-shifted and concurrent; the obtained data also showed a relevant decrease of the time needed to kill the larvae. Particularly, the maximum mortality rate, corresponding to 79% already at 16 h, was observed with the highest concentrations (0.564 µg/mL of *B. thuringiensis* and 8 × 10^2^ IJs of *S. carpocapsae*) in the concurrent trials. This study, conducted by laboratory tests under controlled conditions, is a good starting point to develop a further application step through field studies for the control of *D. suzukii*.

## Introduction

The biological control of many invasive species of insects is carried out with bio-insecticides^1,2^ such as *Bacillus thuringiensis* (Bt), entomopathogenic nematodes (EPN), entomopathogenic fungi or parasitoid wasps^3–8^. Their effectiveness in limiting the spread of phytophagous organisms depends on several factors such as target specificity, administration method and timing, climatic and environmental conditions^9,10^.

Among the bio-insecticides, Bt and EPN are the most used against several insect orders. Bt is a Gram-positive microorganism usually administered by spraying on foliage in the pests infestation area. When ingested by insect larvae during feeding, it sporulates into the intestine producing toxins (delta-toxins). These molecules (Cry and Cyt families) are specific to receptors present on the intestinal epithelium and their binding induces irreversible damage to the epithelial cells, forming pores or lysing the target membranes of the midgut^11,12^.

EPN, as *Steinernema* spp. and *Heterorhabditis* spp., are also used by spraying aqueous suspensions on infested crops. Unlike Bt, nematodes are not ingested but can reach and enter the target insect autonomously. Their lethal action is based on the synergism between the EPN, *Steinernematidae* spp*.* or *Heterorhabditidae* spp., and the symbiotic bacteria, *Xenorhabdus* spp. or *Photorhabdus* spp. respectively, living in their gut: the third stage of the nematode, also named infective juvenile (IJ) stage, penetrates the insect body and reaches the hemocoel cavity of the host where symbiotic bacteria are released and, due to their proliferation and toxins secretion, affect the physiology of the larvae up to septicemia and death^13,14^. The lethality and effectiveness of EPN is closely related to its interaction with the target and the possibility of reproduce itself undisturbed inside the host's body^4,15,16^.

In general, both Bt and EPN have weaknesses in their efficacy, such as possible drying caused by rapid climatic variations, and, in particular for Bt, UV sensitivity and a narrow range of specificity^17,18^; therefore, a reduction in the time lapse between administration and relevant mortality of the target insect population, would help to preserve the integrity of the crops affected by the infestation.

The fly *Drosophila suzukii* (Matsumura, 1931) (Diptera: Drosophilidae), also known as spotted wing drosophila, is an invasive pest endemic to South East Asia, which has recently invaded western countries^19^, threatening both European and American fruit industry. *D. suzukii* is one of the most important emerging pests of soft-skinned fruit such as raspberries, blackberries, strawberries, blueberries, cherries, grapes, and peaches^20^. The adult female lays her eggs into the fruit through the ovipositor by perforating the skin, and the larvae develop by feeding on the fruit flesh^21^.

The current control of *D. suzukii* is mainly based on pesticides, but treatments carried out on the orchards, in addition to the known negative impact on the environment, are also troublesome due to the different regulatory framework between producer countries and international export markets^22^. It is therefore advisable to implement and improve biological control techniques. Various bio-insecticides have been tested against *Drosophila* spp*.*, such as parasitoids, entomopathogenic fungi, Bt and EPN such as *Steinernema carpocapsae* (Sc), some of them have exhibited a moderate efficiency in limiting the expansion of this pest^23–26^.

In laboratory assays, the application of Bt or EPN produces a relevant mortality of the target subjects in times ranging from 48 to 72 h after administration^25,27^. Conversely, in field treatments the times are longer by far^28^.

In this work we investigated the effects of Bt subsp. *kurstaki* and Sc, administered individually or in combination, on *D. suzukii* larvae. The individual administration of these bio-insecticides could allow to eliminate a large part of the *D. suzukii* population, but the time required to obtain the result is such that damage to crops is relevant. For this reason, we designed laboratory tests that, using both Bt and Sc in the same administration (concurrent or time-shifted), would allow to evaluate the combined effects of these bio-insecticides aimed to a possible reduction of the time needed to eradicate the *D. suzukii* population. We carried out a controlled experimental investigation using administration techniques that were as much compatible as possible with field tests, to ensure that the obtained data reflected natural behavior of target insect and entomopathogens. For this purpose, we used a breeding and feeding system for *D. suzukii* that allowed the larvae to feed properly by ingesting Bt and supported the EPN locomotion for the active search of the host.

## Results

### Evaluation of the efficacy of *B. thuringiensis* on *D. suzukii* larvae

**Figure 1:**
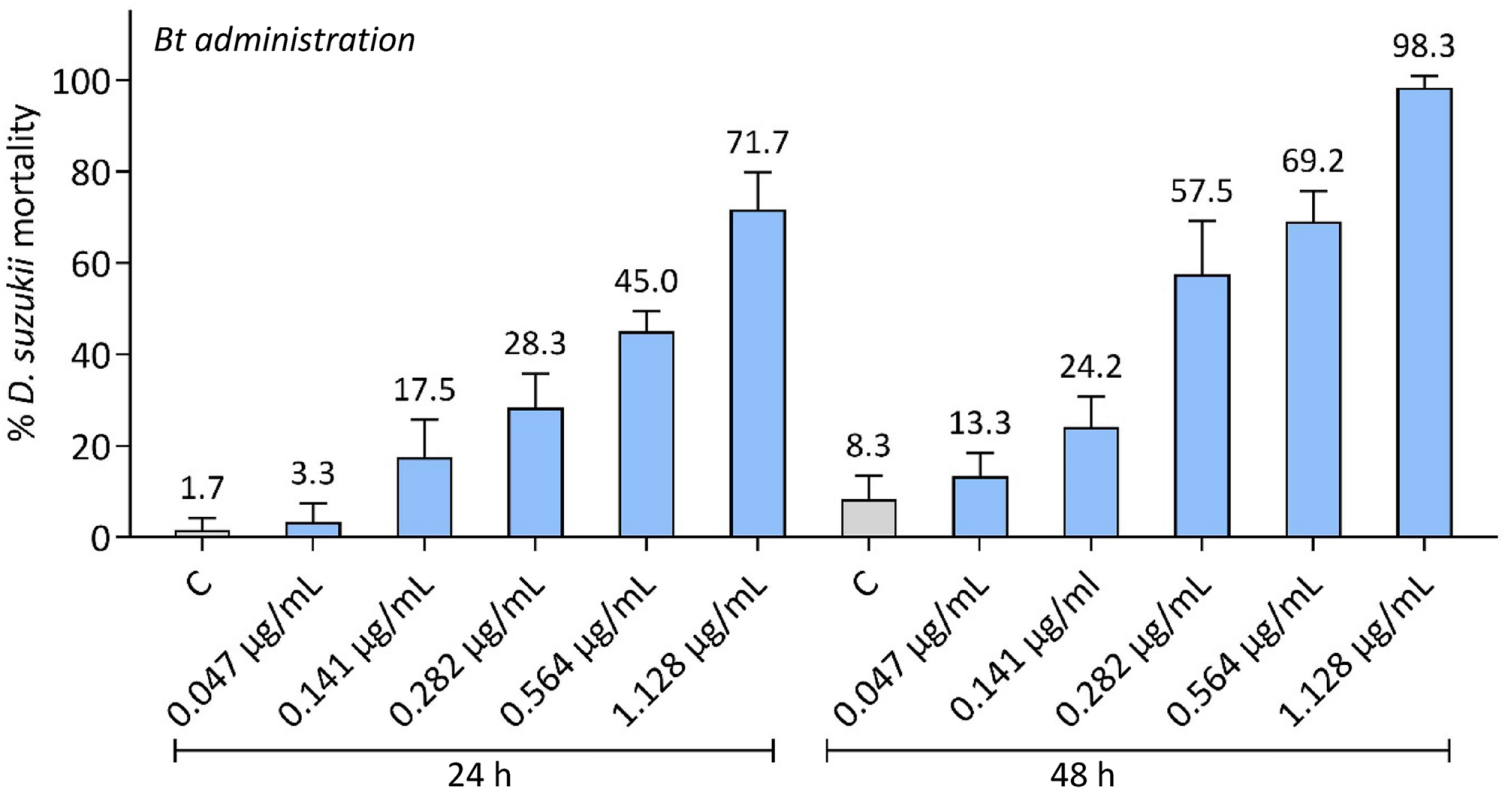
Effects of various concentrations of *B. thuringiensis* (Bt) administered to L1 stage of *D. suzukii* larvae. Results were recorded 24 and 48 h post-treatment. A relevant mortality was observed, at 24 h, with the highest concentration (1.128 μg/mL), and at 48 h, also with 0.564 μg/mL. The trend in the graphs clearly indicates that the efficiency of the bacteria is dependent on the administered concentration. *C* control.

*D. suzukii* larvae at early larval stage (L1) were treated with five increasing concentrations of Bt and mortality was monitored at 24 and 48 h. Infections were carried out with the agar-trap method, Bt was added to the soft agar at concentrations ranging from 0.047 to 1.128 μg/mL. The obtained data showed a significant concentration and time-dependent efficacy (Fig. 1).

Larvae mortality increased with both concentration (ANOVA, F_5,72_ = 138.8, p < 0.001) and treatment time (ANOVA, F_1,72_ = 70.5, p < 0.001). Two-way ANOVA results also showed a significant interaction between these two factors (F_5,72_ = 4.3, p = 0.002). At both 24 and 48 h, larvae mortality between concentrations were significantly different (Tukey test, p < 0.005, Table S1), except for the lowest concentration (0.047 μg/mL) and the control or 0.141 μg/mL (only at 48 h), and between 0.282 μg/mL and 0.141 (only at 24 h) or 0.564 μg/mL. Also, comparisons within the same concentration at the two monitored time intervals were statistically significant (Tukey test, p < 0.05, Table S1), except for the two lowest concentrations (0.047 and 0.141 μg/mL). The highest Bt concentration (1.128 μg/mL) resulted in 71.7% and 98.3% of mortality, at 24 and 48 h, respectively. At 48 h, a larvae mortality higher than 50% was observed even at lower concentrations, 0.282 and 0.564 μg/mL (corresponding to 1.41 × 10^–2^ and 2.82 × 10^–2^ μg/cm^2^).

**Figure 2:**
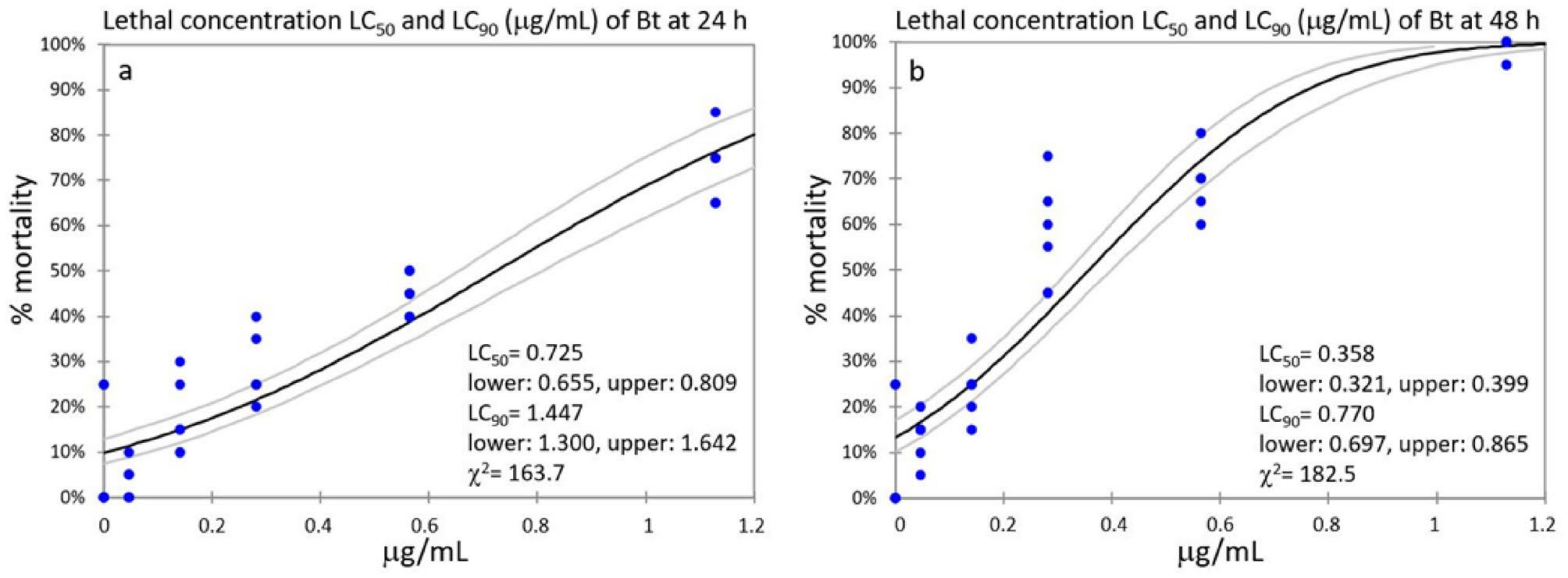
Bt lethal concentrations (LC_50_ and LC_90_), at 24 h (**a**) and 48 h (**b**), were calculated by Probit analysis with 95% confidence intervals.

Bt lethal concentrations were 0.725 μg/mL (LC_50_) and 1.447 μg/mL (LC_90_) at 24 h (Fig. 2a), and 0.358 μg/mL (LC_50_) and 0.770 μg/mL (LC_90_) at 48 h (Fig. 2b). The LC_50_ median value between 24 and 48 h was 0.542 μg/mL.

### Evaluation of the efficacy of *S. carpocapsae* on *D. suzukii* larvae

**Figure 3:**
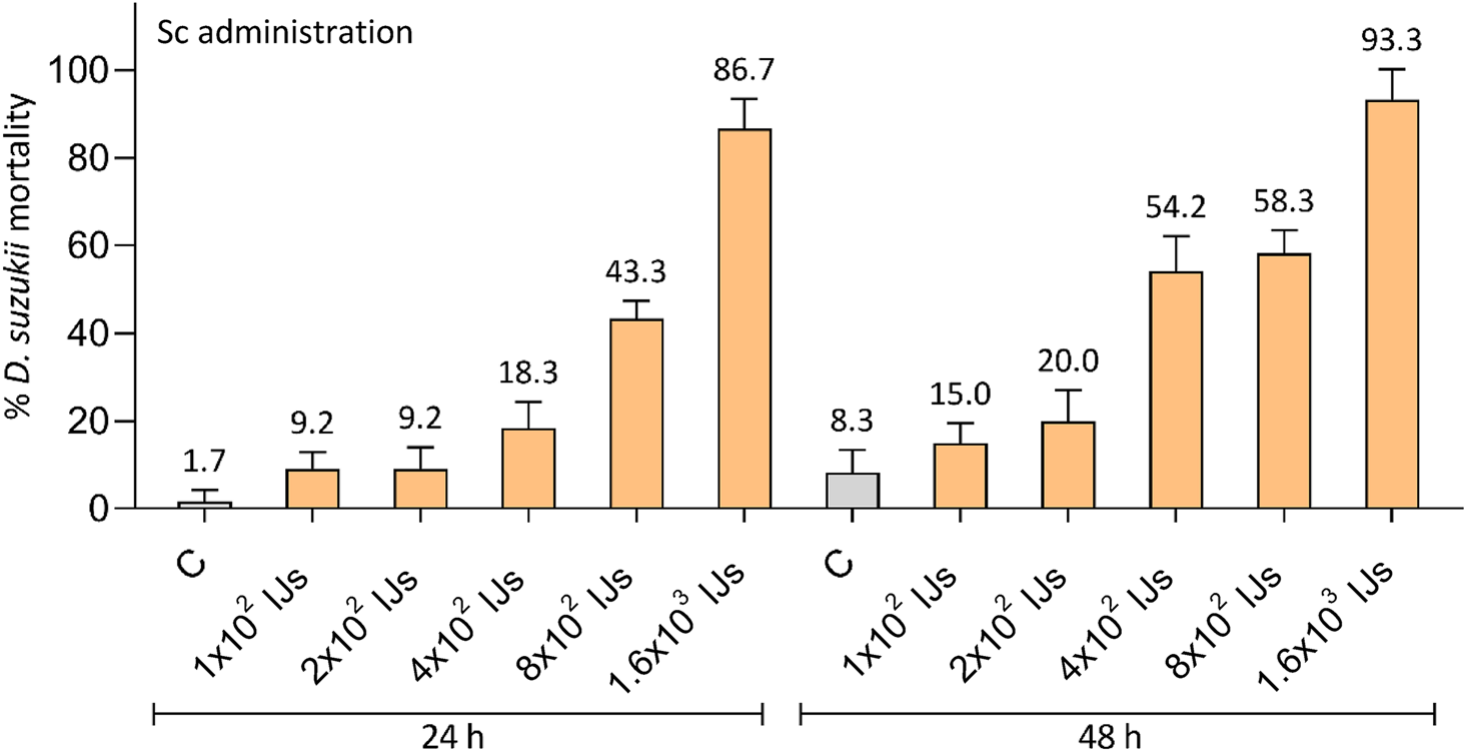
Effects of various amounts of *S. carpocapsae* (Sc) administered to L1 stage of *D. suzukii* larvae. Mortality rates were recorded at 24 and 48 h after administration. A concentration-dependent efficacy was observed, though relevant only with the highest number of Sc (1.6 × 10^3^ IJs), at both 24 and 48 h. *C* control.

Sc in aqueous suspension were added to *D. suzukii* larvae by layering on the surface of the agar traps. Administered Sc ranged from 1 × 10^2^ to 1.6 × 10^3^ IJs (Fig. 3).

As observed for Bt, also for Sc, the efficacy was related to the increase in concentration (ANOVA, F_5,72_ = 134.1, p < 0.001) over time (ANOVA, F_5,72_ = 37.5, p < 0.001). However, two-way ANOVA results showed a lower significant interaction between these two factors (F_5,72_ = 2.4, p = 0.045). At 24 h, the mortality of all treatments was significantly different from the control, and the two highest amounts (8 × 10^2^ and 1.6 × 10^3^ IJs) differed significantly among them and from the lower concentrations (Tukey test, p ≤ 0.01, Table S2). At 48 h, the difference between concentrations increased (Tukey test, p < 0.05, Table S2), except between 1 × 10^2^ IJs and control or 2 × 10^2^ IJs, and between 4 × 10^2^ and 8 × 10^2^ IJs. Differently from Bt, comparisons within the same concentration at the two monitored time intervals were not statistically significant (Tukey test, p > 0.05, Table S2), except for the intermediate amount, 4 × 10^2^ IJs (Tukey test, p = 0.0002, Table S2). A high (> 85%) performance of Sc was observed only at the highest amount (1.6 × 10^3^ IJs) at both 24 and 48 h (Fig. 3). However, such as for Bt, a larvae mortality higher than 50% was observed even at lower amounts, 4 × 10^2^ and 8 × 10^2^ IJs (corresponding to 20 and 40 IJs/cm^2^), at 48 h.

**Figure 4:**
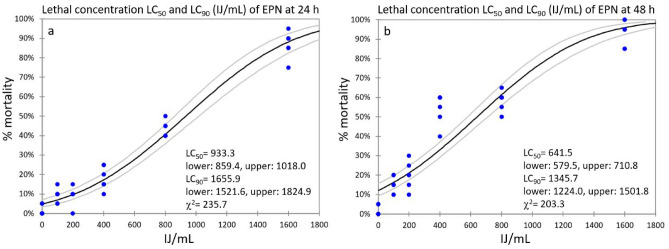
Sc lethal concentrations (LC_50_ and LC_90_), at 24 h (**a**) and 48 h (**b**), were calculated by Probit analysis with 95% confidence intervals.

Sc lethal concentrations were 933.3 IJs/mL (LC_50_) and 1655.9 IJs/mL (LC_90_) at 24 h (Fig. 4a), and 641.5 IJs/mL (LC_50_) and 1345.7 IJs/mL (LC_90_) at 48 h (Fig. 4b). The LC_50_ median value between 24 and 48 h was 787.4 IJs/mL.

### Effects of *B. thuringensis* on *S. carpocapsae*

**Figure 5:**
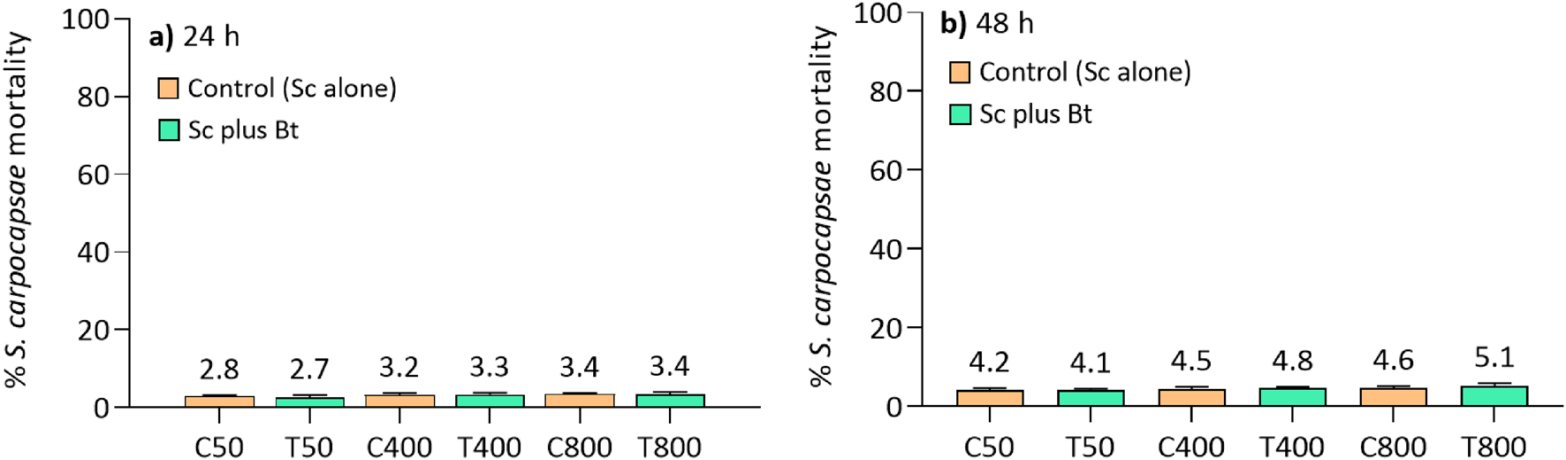
Evaluation of the potential adverse effects of Bt (0.564 μg/mL) on Sc (5 × 10, 4 × 10^2^ and 8 × 10^2^ IJs) viability at 24 h (**a**) and 48 h (**b**). The mortality of Sc in all assays did not exceed about 3% (24 h) and 5% (48 h) and was comparable to the controls with Sc alone. These assays were carried out before the combined administration of the two bio-insecticides. C: control, Sc alone; T: treatment, Sc plus Bt.

We carried out in vitro assays to ascertain possible adverse effects of the presence of Bt on Sc viability. Bt (0.564 μg/mL) were co-incubated in agar traps with Sc (5 × 10, 4 × 10^2^ and 8 × 10^2^ IJs) and the percentage of dead nematodes was evaluated at 24 and 48 h (Fig. 5). The mortality of the treatments did not significantly differ from that of the relative controls (*t* test, p > 0.05) and not exceed about 3% and 5% at 24 (Fig. 5a) and 48 h (Fig. 5b) respectively. Thus, these results excluded a possible toxicity of Bt against Sc.

### Effects of the time-shifted administration of *B. thuringiensis* and *S. carpocapsae*

**Figure 6:**
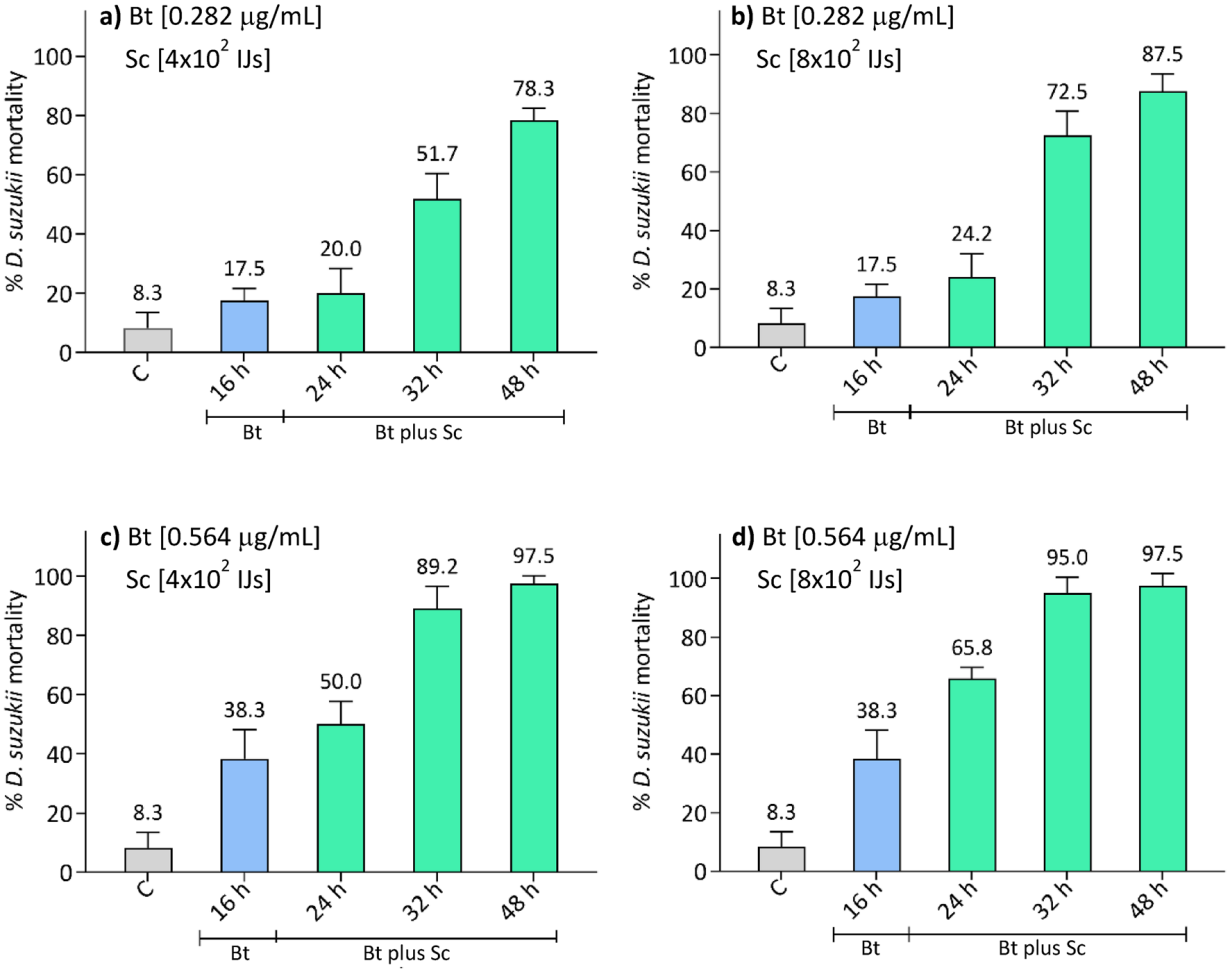
Mortality of *D. suzukii* after time-shifted administration of Bt at t_0_, plus Sc after 16 h. Graphs from (**a**) to (**d**) show the effects of different concentrations of the two entomopathogens at 16, 24, 32 and 48 h. Trials show an improvement in the effectiveness of the combination that mainly depends on the increase in Bt concentration. However, all results show a clear enhancement of the efficacy compared to assays with single administration of entomopathogens (Figs. 1 and 3). *C* control.

We carried out combined trials with different concentrations of Bt (0.282 and 0.564 μg/mL) and numbers of Sc (4 × 10^2^ and 8 × 10^2^ IJs). Bt was administered at t_0_ and the Sc addition was performed with a delay of 16 h (t_16_). Figure 6 shows the effects of 0.282 μg/mL of Bt and 4 × 10^2^ IJs (Fig. 6a) or 8 × 10^2^ IJs (Fig. 6b) of Sc. A further combined assay was performed increasing the Bt concentration to 0.564 μg/mL and administering Sc at the same two amounts, 4 × 10^2^ IJs (Fig. 6c) or 8 × 10^2^ IJs (Fig. 6d).

Two-way ANOVA results were significant for both factor treatment (F_4,120_ = 144.5, p < 0.001) and factor time (F_3,120_ = 109.0, p < 0.001), and for their interaction (F_12,120_ = 7.7, p < 0.001). The mortality of treatments was always significantly higher than that of controls (Tukey test, p < 0.05, Table S3). The increase in the number of added nematodes, maintaining a constant concentration of Bt, did not result in a significant improvement in the lethal effect against *D. suzukii* larvae (Tukey test, p > 0.05, Table S3). The obtained data showed instead that, using a higher concentration of Bt, an increase of mortality occurred at 24 h post-treatment a mortality equal to or greater than 50% was observed and almost all *D. suzukii* larvae were dead after 48 h. From 24 h, the mortality of Bt plus Sc combination 0.282 μg/mL–4 × 10^2^ IJs was significantly lower than that of the combinations 0.564 μg/mL–4 × 10^2^ IJs and 0.564 μg/mL–8 × 10^2^ IJs (Tukey test, p < 0.01, Table S3). The mortality of Bt plus Sc combination 0.282 μg/mL–8 × 10^2^ IJs was instead significantly lower than that of the highest combination 0.564 μg/mL–8 × 10^2^ IJs, at 24 and 32 h (Tukey test, p < 0.01, Table S3). Within all combinations, the mortality significantly increased after 24 h (Tukey test, p ≤ 0.005, Table S3), except for the combinations with the highest Bt concentration, 0.564 μg/mL–4 × 10^2^ IJs and 0.564 μg/mL–8 × 10^2^ IJs, between 32 and 48 h.

### Effects of the concurrent administration of *B. thuringiensis* and *S. carpocapsae*

**Figure 7:**
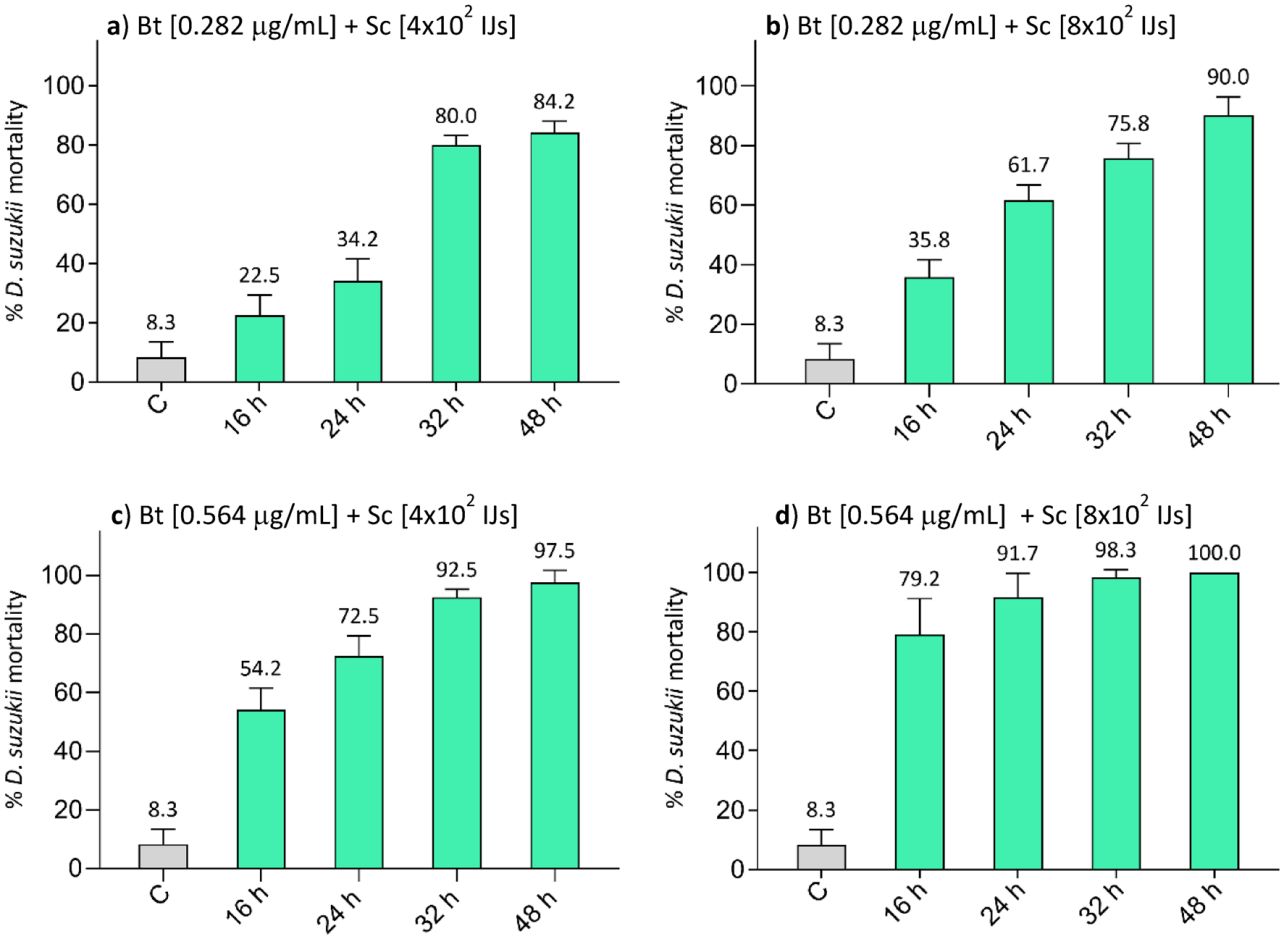
Simultaneous administration of Bt and Sc to *D. suzukii* larvae. Both Bt and Sc were added to the agar traps at t_0,_ and larvae mortality was recorded at 16, 24, 32, 48 h. The greater effectiveness of this treatment is clear compared to both individual (Figs. 1 and 3) and time-shifted (Fig. 6) administrations. In these assays, either the increase in Bt or Sc concentrations has a positive effect on the efficacy of the treatment, and, except for the lowest combination (**a**), a reduction in the time lapse between administration and mortality was observed. With the highest combination (**d**), a relevant mortality rate, about 80%, was already observed 16 h after the start of the trials and reached 100% at 48 h. *C* control.

A second set of trials was carried out by concurrent administration of the two entomopathogens. Bt (0.282 and 0.564 μg/mL) and Sc (4 × 10^2^ and 8 × 10^2^ IJs) were both added to the agar traps at t_0_ (Fig. 7).

Even in this case, two-way ANOVA results were significant for both factor treatment (F_4,120_ = 221.3, p < 0.001) and factor time (F_3,120_ = 63.1, p < 0.001), and for their interaction (F_12,120_ = 5.5, p < 0.001). The mortality of treatments was always significantly higher than that of controls (Tukey test, p < 0.001, Table S4). The increase in the number of added nematodes resulted in a significant increase of *D. suzukii* larvae mortality only at 24 h for both the tested Bt concentrations and also at 16 h for the highest Bt concentration (0.564 μg/mL) (Tukey test, p < 0.05, Table S4). The mortality of Bt plus Sc combination 0.282 μg/mL–4 × 10^2^ IJs was significantly lower than that of the two combinations with the highest concentration of Bt (0.564 μg/mL–4 × 10^2^ IJs and 0.564 μg/mL–8 × 10^2^ IJs) at all the monitored times (Tukey test, p < 0.05, Table S4), except for 0.564 μg/mL–4 × 10^2^ IJs, at 32 h. Significant differences were also observed at all the monitored times for the combination 0.282 μg/mL–8 × 10^2^ IJs compared to the combination 0.564 μg/mL–8 × 10^2^ IJs (Tukey test, p < 0.001, Table S4). Within all Bt plus Sc combinations, the mortality significantly increased over time in most cases (Tukey test, p < 0.05, Table S4), except for the combination 0.564 μg/mL–8 × 10^2^ IJs that caused the mortality of almost all *D. suzukii* larvae (90–100%) already at 24 h after treatment. This combination led to a relevant mortality (approximately 80%) also at 16 h after treatment.

## Comparison between single and combined treatments

**Figure 8:**
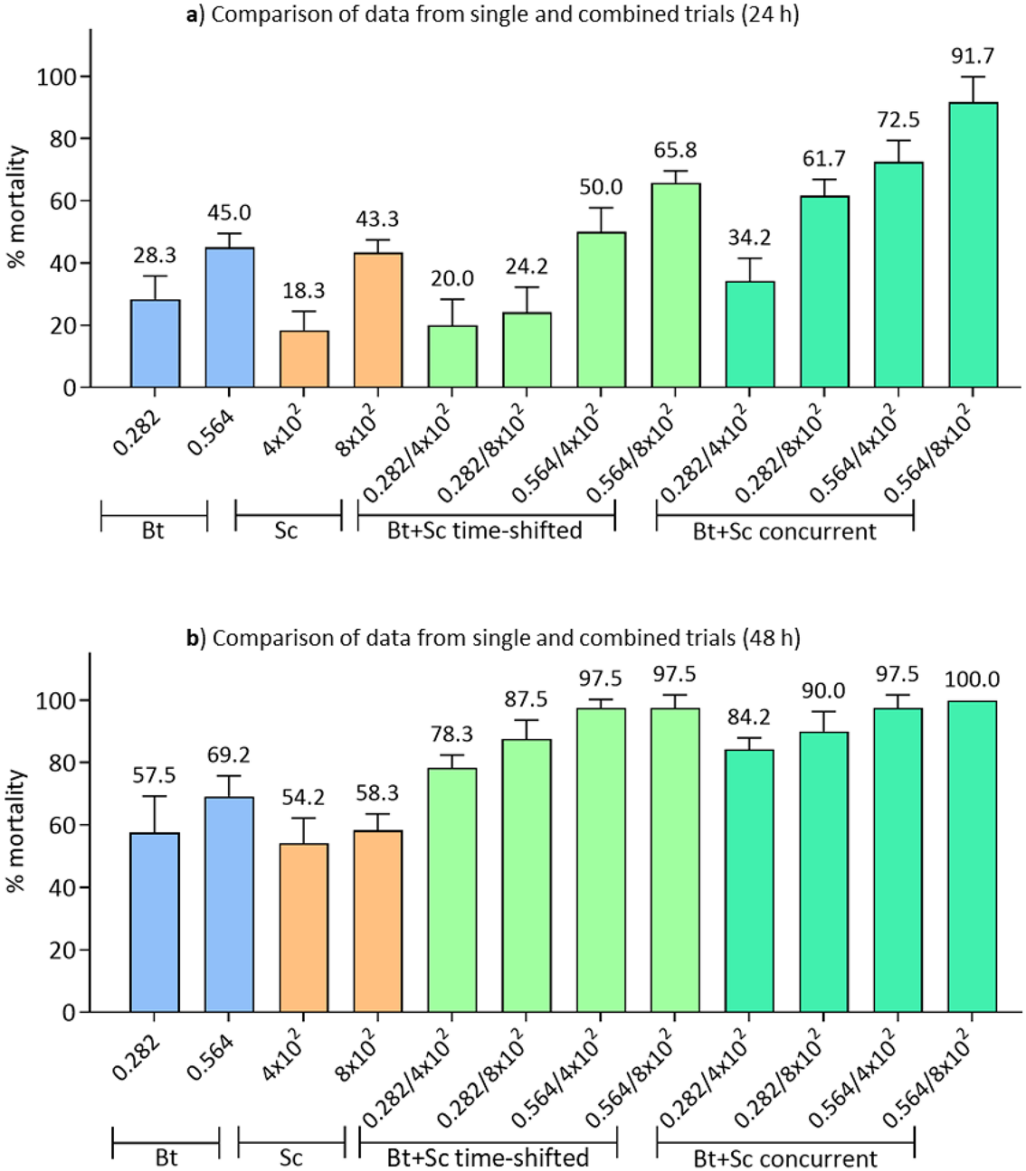
Cross assessment of the results obtained in single and combined administrations at 24 and 48 h. The graphs allow a direct comparison of the data obtained in the different trials showing the general improvement in the effectiveness of bio-insecticides when used in combination against *D. suzukii* larvae. A significant increase in mortality induced by the concurrent administration of Bt and Sc is evident. Bt concentration is expressed in μg/mL, Sc administration is expressed as total number of IJs.

Results of the comparison between single and combined administrations at 24 h (ANOVA, F_11,72_ = 49.4, p < 0.001) and 48 h (ANOVA, F_10,66_ = 33.3, p < 0.001) are shown in Fig. 8 and Table S5.

Significant difference of larvae mortality between single treatment with Bt and Sc was observed only at 24 h between 0.564 μg/mL Bt concentration and 4 × 10^2^ IJs of Sc (Tukey test, p = 0.0002) (Fig. 8a). Both combined trials (time-shifted and concurrent administration) revealed more efficient than trials with single bio-insecticide, particularly at high concentrations of both Bt and Sc and after 48 h (Tukey test, p < 0.05) (Fig. 8b). Significant differences (Tukey test, p < 0.01) in the efficacy of the combined use of the two entomopathogens between time-shifted and concurrent administration trials were recorded at 24 h (except for the lowest combination 0.282 μg/mL–4 × 10^2^ IJs) but not at 48 h (Fig. 8a,b). These results confirmed that the concurrent presence of the two entomopathogens does not affect their efficacy.

## Discussion

Introduced through human activities, such as travels and transcontinental trade, and supported by climate change, several invasive species can settle in habitats where formerly they could not survive. The presence of allochthonous species can negatively affect native species reducing biodiversity, represent a threat to human health and, in the case of phytophagous insects, cause serious damage to the agroeconomic system^29^. Characterized by rapid spread and enormous impact, an invasion like that of *D. suzukii* has few precedents. This species is thus becoming a model of study in the biology of alien species and for the development of pest management techniques^30^.

The protection of crops from *D. suzukii* invasion is mainly conducted by means of insecticides^31^ which, as known, are unselective, in many cases remain in the environment and, if persistent on fruit, are harmful to human and animal health^32^. In addition, their efficiency can be reduced by weather events such as rainfall, and their prolonged use may induce resistance phenomena in target insects. Moreover, the preference of *D. suzukii* for ripening fruit requires that any treatment be carried out close to the harvesting, which inevitably means that insecticide residues can remain on the marketed fruit^33^. Alternatively to synthetic chemicals, natural compounds that can act as repellents, toxicants by contact or ingestion, and overall deterrents, have been tested mainly against *D. suzukii* adults^34^. All these control procedures have positive aspects, such as low cost, sometimes good effectiveness, and ease of use for farmers, but, as mentioned, their massive use affects environment and organisms at various levels. Considering the limits of the chemical control, several studies have addressed the problem of *D. suzukii* management through the development and improvement of biological control methods. However, given the recent introduction of this insect in Europe and America specific projects in these areas are still limited. Until now, only the main entomopathogens commonly used in biological control, have been tested against *D. suzukii*^35^*.*

Several serovars of Bt were tested at different concentrations on *D. suzukii*. Cossentine et al.^27^ described the results of the assays with 22 serotypes of Bt, and among them, only few are effective against the target dipteran. In particular, the serovars *thuringiensis, kurstaki, thompsoni, pakistani,* and *bolivia* show an effectiveness of more than 75% on first stage larvae. The mortality rates, however, are related to a dietary administration of at least 10^8^ spores/mL, below this concentration the insecticidal activity is not relevant. Moreover, the efficacy on adult individuals is extremely low, only the serovar *thuringiensis* induces a mortality of about 44%. In the literature, data on the low effects of serovar *israelensis* on this fly are also reported^36^. The data obtained from our trials, carried out with the serovar *kurstaki*, agree with Cossentine et al.^27^, demonstrating that only high concentrations of the bacterium produce significant effects on larvae in a relatively short time (24–48 h).

As regards the insecticidal activity of EPN, the literature includes several studies carried out in laboratory or field with applications on plants and soil. The data collected using different species of nematodes, such as *S. carpocapsae* (Sc), *S. feltiae*, *S. kraussei*, *H. bacteriophora*^25,37–39^ and the rare species *Oscheius onirici*^40^, are quite promising but, as observed for Bt, also for EPN high concentrations are required, and their effectiveness is very variable between laboratory and field studies. In accordance with previous results^29^, our current data indicate a good efficacy of Sc: treatments with an amount of 1.6 × 10^3^ IJs (corresponding to 80 IJ/cm^2^) resulted in a larvae mortality above 98%, 48 h post-treatment.

Since the reproduction of *D. suzukii* occurs by spawning in the mesocarp of the fruit, the technical limit of the application of bio-insecticides such as Bt or EPN cannot be disregarded. Larvae development is protected by the fruit pulp and this may be one of the causes of the low effectiveness of Bt in field applications. As known, to be effective, Bt must be ingested and should therefore reach the larva by penetrating inside the fruit; perhaps a timely spraying of the crops before oviposition could theoretically promote the penetration of microorganisms thanks to the drive of ovipositor. Based on these considerations, it is reasonable to assume that coupling Bt with EPN, which, being motile, can actively reach the targets, could significantly improve the effectiveness of *D. suzukii* control methods in the field.

**Figure 9:**
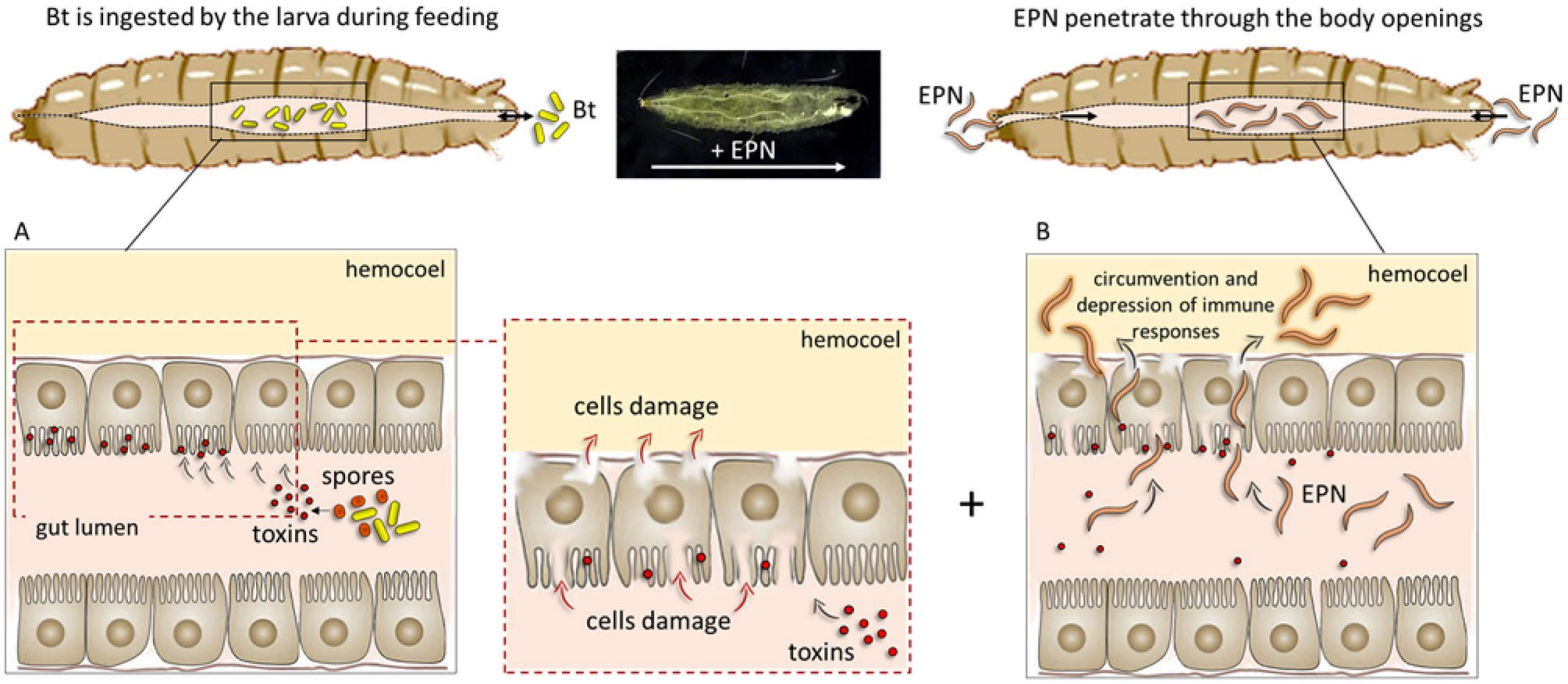
A possible model of the effects induced by the combined administration of *B. thuringiensis* (Bt) and *S. carpocapsae* (EPN) to *D. suzukii* larvae. The presence of *B. thuringiensis*, ingested during feeding by *D. suzukii* larvae, could facilitate and speed up the passage of *S. carpocapsae* from the intestine to the hemocoelic cavity. Bt toxins, produced as parasporal inclusions, are activated in the intestinal lumen of the larva. Active toxins interact with the membrane receptors of the intestinal epithelium and are responsible for the formation of pores that alter the physiology of the cells leading to their lysis. The resulting epithelial lesions could provide an easy access route for EPN to other body regions of the larvae.

The simultaneous application of two control agents, with different modes of action, may result in additive or complementary effects. In particular, the tissue damage inflicted by Bt to the intestinal epithelium could facilitate the access of nematodes in the passage from the gut to the hemocoel (Fig. 9). When in the hemolymph, the nematode and its symbionts implement strategies of immunoevasion and immunodepression of host immune responses^4,41,42^, leading to a drastic and more rapid physiological alteration that results in the death of the target insect in a shorter time.

As previously reported, and supported by literature, it is desirable that studies on biological control of *D. suzukii* will be increased and possibly new strategies for the use of bio-insecticides will be tested. In this perspective, we started a project aimed at assessing whether the association of entomopathogenic organisms and microorganisms is a promising strategy, capable of making safer and faster measures to control the diffusion of insect pests. Besides this, a need to reduce Bt concentrations used in field application has arisen from recent observations on the possible insecticidal effect on non-target insect populations, caused by intensive use and consequent bioaccumulation of the bacterium in crop applications^43^. It is also known that high concentrations of Bt serovars which produce the cry1Ba1 and 1 beta-exotoxin toxins, are toxic not only to several dipterans but also to mammals^44,45^.

Even when using EPN, limitations have been highlighted: such as with Bt, they are susceptible to runoff in case of rainfall, have a significant sensitivity to drying at high temperatures, and, depending on the species, are more or less effective in certain environmental conditions as well as susceptible to the presence of pesticides^46,47^.

On the basis of these considerations and the relevant literature, and after excluding adverse effects on the nematode induced by the presence of Bt, we conducted the assays with combinations of Bt and Sc, administering them both simultaneously and time-shifted. According to our data, the mortality of *D. suzukii* larvae increases from < 45%, in single administration of Bt or Sc, to 65.8%, in the case of combined time-shifted administration (Bt: 0.564 μg/mL and Sc: 8 × 10^2^ IJs) at 24 h. These effects are even more significant in the case of trials carried out by simultaneous administration; in this case the mortality within 24 h rises to 91.7%. At 48 h post-treatment, the data show a lethality that rises to 78.3% using the lowest concentrations of the two entomopathogens (Bt: 0.282 μg/mL and Sc: 4 × 10^2^ IJs), until reaching the total mortality of the larvae with the highest concentrations (Bt: 0.564 μg/mL and Sc: 8 × 10^2^ IJs).

The data at 24 h indicate a clear concentration-dependence of Bt, which at the highest concentrations is extremely effective in all the combined tests. Sc seems to be mainly influenced by the incubation time with the larvae. This could be explained by the different mechanism of action and intake of the two bio-insecticides: Bt is ingested rapidly by the larva during feeding, while Sc must actively seek and penetrate the host; thus, both time and concentration could contribute to a greater effectiveness of the nematodes. The administration of Bt at t_0_ in both combined trials is justified by the specific action at the intestinal level of this bacterial strain^48^ since the damage and possible lesions at the level of the intestinal epithelium can promote the rapid passage of Sc with its symbionts to the hemocoelic cavity (Fig. 9). Moreover, the altered physiology of the larva, induced by Bt, makes the insect more permissive to the action of the nematode. Conversely, anticipating the administration of Sc would not support the action of Bt, as the debilitated larvae may not actively feed, therefore not ingesting an enough number of bacteria.

Our data agree with the literature describing the increased efficacy of the use of entomopathogen combinations. Bt plus EPN combination was used against other insect species, such as the Coleoptera *Cyclocephala hirta* and *Cyclocephala pasadenae*^49^, the Lepidoptera *Spodoptera exigua, Autographa gamma* and *Plutella xylostella*^50,51^, and the Diptera *Tipula paludosa*^52^ and *Culex pipiens*^53^.

The results of the application of different combined formulations, such as entomopathogens with pesticides, or with products of natural origin, suggest that their concurrent effects improve the effectiveness compared to single uses^54–56^. Also, the combination of entomopathogens with pesticides could reduce the needed amount of the chosen pesticide. This kind of trials always requires checking for possible adverse effects of the chemical on the viability of organisms or microorganisms coupled in the formulation and during the administration^57–59^.

Our results obtained by the simultaneous administration of Bt and Sc to *D. suzukii* larvae show a significant increase in the efficacy and a substantial reduction in the time of killing of the larvae. A reduction of the time lapse between administration and effects is particularly important for the control of *D. suzukii*: since the larvae of this fly prefer fruit close to ripening, so just before harvesting and sale, the speed of neutralization is essential to avoid excessive damage to the harvest that would affect its marketing. Moreover, the combined use of Bt and Sc may allow to reduce the amount of both the bio-insecticides applied in the field, limiting possible ecological issues and maybe improving the cost–benefit ratio.

The trials performed in the laboratory are obviously not transposable in an immediate intervention in the field but provide an essential basis for the possible improvement of methods and technologies that can be used in the field. This is particularly stimulating when the test results, which prelude possible trials on crops, are unequivocally encouraging. However, it is important to consider that the variability of the protocols used by the investigators in laboratory, semi-field and field tests, and the consequent lack of standardized protocols in the experimentation, often make difficult a correct data comparison. Besides this, the great individual variability in the physiology of the organisms used, as both bio-insecticides and target organisms, should be accounted for.

To our knowledge, this is the first work carried out on *D. suzukii* that assesses the possible combination of *B. thuringiensis* and *S. carpocapsae*. The data obtained represent a good starting point to improve the methods of control of the spread of this pest and can stimulate further studies aimed at an extensive screening of the possible combined administrations, fundamental for a future conscious and safe use of biological control methods.

## Methods

### Chemicals and instruments

All reagents were supplied by Sigma Chemicals (St. Louis, MO, USA), ICN (ICN Biomedicals, GmbH), Merck Millipore Ltd. (Tullagreen, Cork, Ireland). Instruments were supplied by Bio-Rad Laboratories (Detroit, MI, USA) and Celbio Spa (Milan, Italy, EU). Centrifugations were carried out with a SIGMA 1–14 (SciQuip Ltd., Newtown, Wem, Shropshire, UK) microcentrifuge and an Eppendorf 5804R (Eppendorf, AG, Hamburg, Germany) centrifuge. All materials, buffers, and solutions were autoclaved or filtered by 0.22 µm Minisart filters (Sartorius, Goettingen, Germany). For microscopy observations, a stereomicroscope SZQ4 (OPTIKA Italy Srl, Ponteranica (BG), Italy) connected to OPTIKA mod. C-HP digital camera was used.

### Target insect and bio-insecticides

*D. suzukii* larvae native to Catalonia (NE Spain) were reared on a specific diet in laboratory^60^ and maintained in a climate chamber at 25 °C, 45% relative humidity (RH), with a 12:12 h photoperiod. For each assay, only healthy larvae at the first stage (L1) of growth were used.

*D. suzukii* susceptibility to entomopathogens was assayed by administration of Bt, subspecies *kurstaki* (strain EG 2348), and Sc (strain B14) collected from urban garden soil in Barcelona (NE Spain) and kindly provided by Prof. Garcia Del Pino (Universitat Autònoma de Barcelona). Sc, supplied at the third IJ stage, were dehydrated and mixed with inert material, then stored at 4 °C to keep parasites in cryptobiosis. Before assays, 2–3 gr of the nematodes were dissolved in dechlorinated water and purified from the inert material. The Sc suspension was layered on a discontinuous sucrose gradient (75–50–25%) and centrifuged at 1000×*g* for 10 min at room temperature; nematodes were recovered at the sucrose 25–50% interface, then washed several times in dechlorinated sterile tap water to remove contaminants, and finally resuspended in water before trials. The number of nematodes used for the trials and vitality control were assessed under a stereomicroscope. Bt was grown in culture media, spores and crystals were isolated as described by Cossentine et al.^27^; their presence was monitored by phase contrast microscopy. Finally, spores and crystals were freeze-dried and, before use, resuspended in filtered sterile tap water.

### *D. suzukii* susceptibility to *B. thuringiensis* and *S. carpocapsae*

As a method of infestation in laboratory trials, we used a Petri agar trap to assess the lethality of the entomopathogens against *D. suzukii* larvae. The agar trap consists of a Petri dish filled with a thin layer of soft agar plus saccharose substrate. The thin layer of agar is transparent enough to allow an easy and accurate evaluation of larvae morphology and vitality. The substrate for the infection was composed of 0.75% agar and 5% sugar in sterile tap water; the solution was heated to 100 °C and then poured (4 mL) into Petri dishes (Ø 5 cm). For Bt trials, when the agar solution was cooled to 37 °C, a suspension containing Bt spores and crystals was added. For Sc assays, Sc suspension (1 mL) was added to the trap when agar was solidified. Twenty *D. suzukii* larvae were transferred to each agar trap, and incubated at 25 °C, 45% RH, in a climate chamber. Assays to evaluate entomopathogens lethality against *D. suzukii* were performed with Bt at concentrations of 0.047, 0.141, 0.282, 0.564, 1.128 µg/mL (corresponding to densities from 2.35 × 10^–3^ to 5.64 × 10^–2^ μg/cm^2^) and Sc numbers equal to 1 × 10^2^, 2 × 10^2^, 4 × 10^2^, 8 × 10^2^, 1.6 × 10^3^ IJs (corresponding to densities from 5 to 80 IJs/cm^2^). *D. suzukii* larvae mortality was monitored at 24 and 48 h after the incubation with entomopathogens, under a stereomicroscope, and larvae lacking movement were considered as dead. All controls were carried out by incubating *D. suzukii* larvae without bio-insecticides.

### *D. suzukii* larvae susceptibility to the combined administration of the entomopathogens

Before assessing the effects of the combined administration of Bt and Sc on *D. suzukii*, we verified a possible harmful effect of Bt formulation on the viability of Sc. Thus, co-incubation assays of the two entomopathogens in agar traps were performed in the absence of the target insect. Briefly, 5 × 10, 4 × 10^2^ and 8 × 10^2^ IJs of Sc were incubated, at 25 °C, in Petri dishes containing 0.564 μg/mL of Bt in agar. The mortality of Sc was evaluated under a stereomicroscope at 24 and 48 h, controls were performed by incubating Sc without Bt.

Combined trials were carried out using Bt at concentrations of 0.282 or 0.564 µg/mL, and 4 × 10^2^ or 8 × 10^2^ IJs of Sc. The entomopathogens were administered together at time zero (t_0_), i.e., concurrent administration, or Sc was administered 16 h after the incubation with Bt, i.e., time-shifted administration. The agar traps, containing 20 *D. suzukii* larvae, were kept at 25 °C, 45% RH, in a climatic chamber. The mortality of the larvae was evaluated at 16, 24, 32 and 48 h, post-incubation. All controls were carried out by incubating *D. suzukii* larvae without bio-insecticides.

### Statistical analysis

For each concentration of Bt and amount of Sc or combination between the two bio-insecticides, as for controls, through the time intervals analyzed (24 and 48 h for single bio-insecticide treatments, and 16, 24, 32, and 48 h for combined administrations), we calculated *D. suzukii* larvae mortality as average ± standard deviation of six replicates. Lethal concentrations (LC_50_ and LC_90_) of Bt and Sc on *D. suzukii* larvae were calculated at 24 and 48 h by Probit analysis with 95% confidence intervals.

For each trial (with single bio-insecticide or with a combination of both bio-insecticides), we applied the two-way analysis of variance (ANOVA) considering treatment and time as variables and the percentages of insect mortality as observations. Before the ANOVA, the percentage data were arcsin-transformed. After the ANOVA we used the Tukey HSD post-hoc test to detect possible significant (p < 0.05) differences of insect mortality between different treatments at the same time and between times within the same treatment. Moreover, we applied the one-way ANOVA followed by the Tukey HSD post-hoc test to detect possible significant (p < 0.05) differences of *D. suzukii* larvae mortality between different single administrations of Bt or Sc and the combined (time-shifted or concurrent) administrations of both bio-insecticides, at 24 and 48 h.

For the assays on the possible effects of Bt on Sc, *t* test was performed to detect significant (p < 0.05) difference between each treatment and the related control at 24 and 48 h.

The statistical analyses were performed using XLSTAT 2014 and PAST 3.09 software.

### Ethics declarations

All applicable international, national and/or institutional guidelines for the care and use of animals were followed.

## Supplementary Information

Supplementary Tables.

## Data Availability

All data are available upon reasonable request to the corresponding author.
