# Supplementary material for: Susceptibility of Drosophila suzukii larvae to the combined administration of the entomopathogens Bacillus thuringiensis and Steinernema carpocapsae
Source: Sci Rep. 2021 Apr 14;11:8149. doi: 10.1038/s41598-021-87469-4 (PMC8046782; doi:10.1038/s41598-021-87469-4)
Supplement: Supplementary file 1 — Supplementary Tables. [file 41598_2021_87469_MOESM1_ESM.docx]

**Susceptibility of *Drosophila suzukii* larvae to the combined administration of the entomopathogens *Bacillus thuringiensis* and *Steinernema carpocapsae*.**

**Maristella Mastore^1^, Silvia Quadroni^2^, Maurizio F. Brivio^1*^**

^1^Lab. of Comparative Immunology and Parasitology, Dept. of Theoretical and Applied Sciences, University of Insubria, Varese, Italy
^2^Lab. of Ecology, Dept. of Theoretical and Applied Sciences, University of Insubria, Varese, Italy

*corresponding author: maurizio.brivio@uninsubria.it, orcid id: 0000-0002-1002-0422

**Supplementary Material**

**Table S1.** Statistical results of Bt assay. Pairwise comparisons of *D. suzukii* larvae mortality by Tukey test between different Bt concentrations (C = control, 0 µg/mL of Bt) at 24 and 48 h, and between different times within the same Bt concentration (expressed as µg/mL). Significant p values (p<0.05) are indicated in bold and highlighted in yellow colour.

|  | **Group 1** | **Group 2** | **Q-value** | **p-value** |
| --- | --- | --- | --- | --- |
| **24 h** | 0.047 | C | 1.3 | 0.95 |
|  | 0.047 | 0.141 | 6.9 | **0.0005** |
|  | 0.047 | 0.282 | 10.0 | **0.0001** |
|  | 0.047 | 0.564 | 14.3 | **0.0001** |
|  | 0.047 | 1.128 | 20.9 | **0.0001** |
|  | 0.141 | C | 8.2 | **0.0002** |
|  | 0.141 | 0.282 | 3.1 | 0.27 |
|  | 0.141 | 0.564 | 7.4 | **0.0003** |
|  | 0.141 | 1.128 | 14.0 | **0.0001** |
|  | 0.282 | C | 11.3 | **0.0001** |
|  | 0.282 | 0.564 | 4.3 | 0.05 |
|  | 0.282 | 1.128 | 10.9 | **0.0001** |
|  | 0.564 | C | 15.6 | **0.0001** |
|  | 0.564 | 1.128 | 6.6 | **0.0009** |
|  | 1.128 | C | 22.2 | **0.0001** |
| **48 h** | 0.047 | C | 3.4 | 0.18 |
|  | 0.047 | 0.141 | 2.5 | 0.52 |
|  | 0.047 | 0.282 | 8.7 | **0.0001** |
|  | 0.047 | 0.564 | 10.8 | **0.0001** |
|  | 0.047 | 1.128 | 19.8 | **0.0001** |
|  | 0.141 | C | 5.9 | **0.003** |
|  | 0.141 | 0.282 | 6.3 | **0.002** |
|  | 0.141 | 0.564 | 8.4 | **0.0002** |
|  | 0.141 | 1.128 | 17.3 | **0.0001** |
|  | 0.282 | C | 12.2 | **0.0001** |
|  | 0.282 | 0.564 | 2.1 | 0.69 |
|  | 0.282 | 1.128 | 11.0 | **0.0001** |
|  | 0.564 | C | 14.2 | **0.0001** |
|  | 0.564 | 1.128 | 9.0 | **0.0001** |
|  | 1.128 | C | 23.2 | **0.0001** |
| **0.047** | 24 h | 48 h | 4.7 | 0.06 |
| **0.141** |  |  | 1.7 | 0.99 |
| **0.282** |  |  | 6.2 | **0.003** |
| **0.564** |  |  | 4.9 | **0.04** |
| **1.128** |  |  | 9.6 | **0.0001** |

**Table S2.** Statistical results of Sc assay. Pairwise comparisons of *D. suzukii* larvae mortality by Tukey test between different Sc concentrations (C = control, 0 IJ/mL of Sc) at 24 and 48 h, and between different times within the same Sc concentration (expressed as IJ/mL). Significant p values (p<0.05) are indicated in bold and highlighted in yellow colour.

|  | **Group 1** | **Group 2** | **Q-value** | **p-value** |
| --- | --- | --- | --- | --- |
| **24 h** | 100 | C | 5.7 | **0.004** |
|  | 100 | 200 | 0.6 | 1.00 |
|  | 100 | 400 | 3.4 | 0.20 |
|  | 100 | 800 | 10.5 | **0.0001** |
|  | 100 | 1600 | 22.8 | **0.0001** |
|  | 200 | C | 5.1 | **0.01** |
|  | 200 | 400 | 3.9 | 0.09 |
|  | 200 | 800 | 11.1 | **0.0001** |
|  | 200 | 1600 | 23.4 | **0.0001** |
|  | 400 | C | 9.1 | **0.0001** |
|  | 400 | 800 | 7.1 | **0.0004** |
|  | 400 | 1600 | 19.4 | **0.0001** |
|  | 800 | C | 16.2 | **0.0001** |
|  | 800 | 1600 | 12.3 | **0.0001** |
|  | 1600 | C | 28.5 | **0.0001** |
| **48 h** | 100 | C | 3.7 | 0.12 |
|  | 100 | 200 | 1.1 | 0.97 |
|  | 100 | 400 | 7.4 | **0.0003** |
|  | 100 | 800 | 8.1 | **0.0002** |
|  | 100 | 1600 | 16.4 | **0.0001** |
|  | 200 | C | 4.8 | **0.02** |
|  | 200 | 400 | 6.3 | **0.001** |
|  | 200 | 800 | 7.0 | **0.0004** |
|  | 200 | 1600 | 15.3 | **0.0001** |
|  | 400 | C | 11.1 | **0.0001** |
|  | 400 | 800 | 0.7 | 1.00 |
|  | 400 | 1600 | 9.0 | **0.0001** |
|  | 800 | C | 11.8 | **0.0001** |
|  | 800 | 1600 | 8.3 | **0.0002** |
|  | 1600 | C | 20.1 | **0.0001** |
| **100** | 24 h | 48 h | 1.8 | 0.98 |
| **200** |  |  | 3.5 | 0.37 |
| **400** |  |  | 7.8 | **0.0002** |
| **800** |  |  | 3.1 | 0.57 |
| **1600** |  |  | 3.1 | 0.56 |

**Table S3.** Statistical results of the assay with time-shifted administration of Bt and Sc (expressed as µg/mL of Bt and IJ/mL of Sc respectively). Pairwise comparisons of *D. suzukii* larvae mortality by Tukey test between different combinations of Bt and Sc concentrations (C = control, 0 µg/mL of Bt and 0 IJ/mL of Sc) at 16, 24, 32 and 48 h, and between different times within the same combination. Significant p values (p<0.05) are indicated in bold and highlighted in yellow colour.

|  | **Group 1** | **Group 2** | **Q-value** | **p-value** |  | **Group 1** | **Group 2** | **Q-value** | **p-value** |
| --- | --- | --- | --- | --- | --- | --- | --- | --- | --- |
| **16 h** | 0.282/400 | C | 4.3 | **0.04** | **32 h** | 0.282/400 | C | 9.2 | **0.0001** |
|  |  | 0.282/800 | 0.0 | 1.00 |  |  | 0.282/800 | 2.1 | 0.19 |
|  |  | 0.564/400 | 4.0 | 0.06 |  |  | 0.564/400 | 5.9 | **0.0007** |
|  |  | 0.564/800 | 4.0 | 0.06 |  |  | 0.564/800 | 6.2 | **0.0002** |
|  | 0.282/800 | C | 4.3 | **0.04** |  | 0.282/800 | C | 12.4 | **0.0001** |
|  |  | 0.564/400 | 4.0 | 0.06 |  |  | 0.564/400 | 3.8 | 0.11 |
|  |  | 0.564/800 | 4.0 | 0.06 |  |  | 0.564/800 | 4.1 | **0.007** |
|  | 0.564/400 | C | 8.4 | **0.0002** |  | 0.564/400 | C | 16.0 | **0.0001** |
|  |  | 0.564/800 | 0.0 | 1.00 |  |  | 0.564/800 | 0.3 | 0.71 |
|  | 0.564/800 | C | 8.4 | **0.0002** |  | 0.564/800 | C | 17.8 | **0.0001** |
| **24 h** | 0.282/400 | C | 4.7 | **0.02** | **48 h** | 0.282/400 | C | 14.6 | **0.0001** |
|  |  | 0.282/800 | 0.9 | 0.96 |  |  | 0.282/800 | 2.1 | 0.59 |
|  |  | 0.564/400 | 5.7 | **0.004** |  |  | 0.564/400 | 5.9 | **0.003** |
|  |  | 0.564/800 | 8.4 | **0.0002** |  |  | 0.564/800 | 6.2 | **0.002** |
|  | 0.282/800 | C | 5.6 | **0.004** |  | 0.282/800 | C | 16.7 | **0.0001** |
|  |  | 0.564/400 | 4.8 | **0.02** |  |  | 0.564/400 | 3.8 | 0.08 |
|  |  | 0.564/800 | 7.5 | **0.0003** |  |  | 0.564/800 | 4.1 | 0.05 |
|  | 0.564/400 | C | 10.4 | **0.0001** |  | 0.564/400 | C | 20.5 | **0.0001** |
|  |  | 0.564/800 | 2.7 | 0.34 |  |  | 0.564/800 | 0.3 | 1.00 |
|  | 0.564/800 | C | 13.1 | **0.0001** |  | 0.564/800 | C | 20.8 | **0.0001** |
| **0.282/400** | 16 h | 24 h | 0.7 | 0.95 | **0.564/400** | 16 h | 24 h | 2.5 | 0.31 |
|  |  | 32 h | 11.1 | **0.0002** |  |  | 32 h | 12.1 | **0.0002** |
|  |  | 48 h | 19.4 | **0.0002** |  |  | 48 h | 15.8 | **0.0002** |
|  | 24 h | 32 h | 10.4 | **0.0002** |  | 24 h | 32 h | 9.5 | **0.0002** |
|  |  | 48 h | 18.7 | **0.0002** |  |  | 48 h | 13.3 | **0.0002** |
|  | 32 h | 48 h | 8.4 | **0.0002** |  | 32 h | 48 h | 3.8 | 0.07 |
| **0.282/800** | 16 h | 24 h | 2.2 | 0.42 | **0.564/800** | 16 h | 24 h | 5.9 | **0.003** |
|  |  | 32 h | 16.5 | **0.0002** |  |  | 32 h | 15.0 | **0.0002** |
|  |  | 48 h | 22.0 | **0.0002** |  |  | 48 h | 16.8 | **0.0002** |
|  | 24 h | 32 h | 14.3 | **0.0002** |  | 24 h | 32 h | 9.1 | **0.0002** |
|  |  | 48 h | 19.8 | **0.0002** |  |  | 48 h | 10.9 | **0.0002** |
|  | 32 h | 48 h | 5.4 | **0.005** |  | 32 h | 48 h | 1.8 | 0.60 |

**Table S4.** Statistical results of the assay with concurrent administration of Bt and Sc (expressed as µg/mL of Bt and IJ/mL of Sc respectively). Pairwise comparisons of *D. suzukii* larvae mortality by Tukey test between different combinations of Bt and Sc concentrations (C = control, 0 µg/mL of Bt and 0 IJ/mL of Sc) at 16, 24, 32 and 48 h, and between different times within the same combination. Significant p values (p<0.05) are indicated in bold and highlighted in yellow colour.

|  | **Group 1** | **Group 2** | **Q-value** | **p-value** |  | **Group 1** | **Group 2** | **Q-value** | **p-value** |
| --- | --- | --- | --- | --- | --- | --- | --- | --- | --- |
| **16 h** | 0.282/400 | C | 5.2 | **0.009** | **32 h** | 0.282/400 | C | 16.8 | **0.0001** |
|  |  | 0.282/800 | 2.4 | 0.44 |  |  | 0.282/800 | 0.9 | 0.97 |
|  |  | 0.564/400 | 5.7 | **0.004** |  |  | 0.564/400 | 3.4 | 0.14 |
|  |  | 0.564/800 | 10.3 | **0.0001** |  |  | 0.564/800 | 6.9 | **0.0005** |
|  | 0.282/800 | C | 7.7 | **0.0002** |  | 0.282/800 | C | 15.9 | **0.0001** |
|  |  | 0.564/400 | 3.2 | 0.18 |  |  | 0.564/400 | 4.3 | **0.04** |
|  |  | 0.564/800 | 7.8 | **0.0002** |  |  | 0.564/800 | 7.8 | **0.0002** |
|  | 0.564/400 | C | 10.9 | **0.0001** |  | 0.564/400 | C | 20.2 | **0.0001** |
|  |  | 0.564/800 | 4.6 | **0.02** |  |  | 0.564/800 | 3.5 | 0.12 |
|  | 0.564/800 | C | 15.5 | **0.0001** |  | 0.564/800 | C | 23.8 | **0.0001** |
| **24 h** | 0.282/400 | C | 6.9 | **0.0006** | **48 h** | 0.282/400 | C | 14.0 | **0.0002** |
|  |  | 0.282/800 | 4.4 | **0.03** |  |  | 0.282/800 | 1.6 | 0.66 |
|  |  | 0.564/400 | 6.2 | **0.002** |  |  | 0.564/400 | 4.5 | **0.02** |
|  |  | 0.564/800 | 11.0 | **0.0001** |  |  | 0.564/800 | - | - |
|  | 0.282/800 | C | 11.3 | **0.0001** |  | 0.282/800 | C | 15.6 | **0.0002** |
|  |  | 0.564/400 | 1.8 | 0.71 |  |  | 0.564/400 | 2.8 | 0.22 |
|  |  | 0.564/800 | 6.6 | **0.0009** |  |  | 0.564/800 | - | - |
|  | 0.564/400 | C | 13.1 | **0.0001** |  | 0.564/400 | C | 18.4 | **0.0002** |
|  |  | 0.564/800 | 4.8 | **0.02** |  |  | 0.564/800 | - | - |
|  | 0.564/800 | C | 17.9 | **0.0001** |  | 0.564/800 | C | - | - |
| **0.282/400** | 16 h | 24 h | 12.8 | **0.0002** | **0.564/400** | 16 h | 24 h | 5.0 | **0.01** |
|  |  | 32 h | 22.5 | **0.0002** |  |  | 32 h | 12.3 | **0.0002** |
|  |  | 48 h | 25.0 | **0.0002** |  |  | 48 h | 17.0 | **0.0002** |
|  | 24 h | 32 h | 9.7 | **0.0002** |  | 24 h | 32 h | 7.2 | **0.0004** |
|  |  | 48 h | 12.2 | **0.0002** |  |  | 48 h | 12.0 | **0.0002** |
|  | 32 h | 48 h | 2.5 | 0.31 |  | 32 h | 48 h | 4.7 | **0.02** |
| **0.282/800** | 16 h | 24 h | 3.2 | 0.15 | **0.564/800** | 16 h | 24 h | 3.6 | 0.06 |
|  |  | 32 h | 13.7 | **0.0002** |  |  | 32 h | 6.1 | **0.002** |
|  |  | 48 h | 18.9 | **0.0002** |  |  | 48 h | - | - |
|  | 24 h | 32 h | 10.5 | **0.0002** |  | 24 h | 32 h | 2.5 | 0.21 |
|  |  | 48 h | 15.8 | **0.0002** |  |  | 48 h | - | - |
|  | 32 h | 48 h | 5.3 | **0.007** |  | 32 h | 48 h | - | - |

**Table S5.** Statistical results of the comparisons by Tukey test of *D. suzukii* larvae mortality detected in assays carried out with single bio-insecticide (Bt or Sc) and with time-shifted (SA) or concurrent (CA) administration of both Bt and Sc (expressed as µg/mL of Bt and IJ/mL of Sc respectively) at 24 and 48 h. Significant p values (p<0.05) are indicated in bold and highlighted in yellow colour.

| **24 h** | **Group 1** | **Group 2** | **Q-value** | **p-value** | **48 h** | **Group 1** | **Group 2** | **Q-value** | **p-value** |
| --- | --- | --- | --- | --- | --- | --- | --- | --- | --- |
|  | 0.282 | 400 | 3.1 | 0.56 |  | 0.282 | 400 | 0.8 | 1.00 |
|  |  | 800 | 4.3 | 0.12 |  |  | 800 | 0.2 | 1.00 |
|  |  | 0.282/400 SA | 2.7 | 0.74 |  |  | 0.282/400 SA | 5.2 | **0.02** |
|  |  | 0.282/800 SA | 1.3 | 1.00 |  |  | 0.282/800 SA | 8.2 | **0.0002** |
|  |  | 0.564/400 SA | 6.1 | **0.003** |  |  | 0.564/400 SA | 13.7 | **0.0002** |
|  |  | 0.564/800 SA | 10.3 | **0.0001** |  |  | 0.564/800 SA | 14.2 | **0.0002** |
|  |  | 0.282/400 CA | 1.8 | 0.98 |  |  | 0.282/400 CA | 6.8 | **0.0007** |
|  |  | 0.282/800 CA | 9.2 | **0.0001** |  |  | 0.282/800 CA | 9.5 | **0.0002** |
|  |  | 0.564/400 CA | 12.3 | **0.0001** |  |  | 0.564/400 CA | 14.2 | **0.0002** |
|  |  | 0.564/800 CA | 20.5 | **0.0001** |  |  | 0.564/800 CA | - | - |
|  | 0.564 | 400 | 7.9 | **0.0002** |  | 0.564 | 400 | 0.8 | 0.31 |
|  |  | 800 | 0.5 | 1.00 |  |  | 800 | 0.2 | 0.76 |
|  |  | 0.282/400 SA | 7.5 | **0.0002** |  |  | 0.282/400 SA | 5.2 | 0.82 |
|  |  | 0.282/800 SA | 6.0 | **0.004** |  |  | 0.282/800 SA | 8.2 | **0.01** |
|  |  | 0.564/400 SA | 1.4 | 1.00 |  |  | 0.564/400 SA | 13.7 | **0.0002** |
|  |  | 0.564/800 SA | 5.6 | **0.01** |  |  | 0.564/800 SA | 14.2 | **0.0002** |
|  |  | 0.282/400 CA | 3.0 | 0.60 |  |  | 0.282/400 CA | 4.1 | 0.15 |
|  |  | 0.282/800 CA | 4.5 | 0.09 |  |  | 0.282/800 CA | 6.8 | **0.0007** |
|  |  | 0.564/400 CA | 7.5 | **0.0002** |  |  | 0.564/400 CA | 11.4 | **0.0002** |
|  |  | 0.564/800 CA | 15.8 | **0.0001** |  |  | 0.564/800 CA | - | - |
|  | 400 | 0.282/400 SA | 0.4 | 1.00 |  | 400 | 0.282/400 SA | 6.0 | **0.004** |
|  |  | 0.282/800 SA | 1.8 | 0.98 |  |  | 0.282/800 SA | 9.0 | **0.0002** |
|  |  | 0.564/400 SA | 9.2 | **0.0001** |  |  | 0.564/400 SA | 14.5 | **0.0002** |
|  |  | 0.564/800 SA | 13.5 | **0.0001** |  |  | 0.564/800 SA | 15.0 | **0.0002** |
|  |  | 0.282/400 CA | 4.9 | **0.05** |  |  | 0.282/400 CA | 7.7 | **0.0002** |
|  |  | 0.282/800 CA | 12.4 | **0.0001** |  |  | 0.282/800 CA | 10.4 | **0.0002** |
|  |  | 0.564/400 CA | 15.4 | **0.0001** |  |  | 0.564/400 CA | 15.0 | **0.0002** |
|  |  | 0.564/800 CA | 23.7 | **0.0001** |  |  | 0.564/800 CA | - | - |
|  | 800 | 0.282/400 SA | 7.0 | **0.0005** |  | 800 | 0.282/400 SA | 5.0 | **0.03** |
|  |  | 0.282/800 SA | 5.6 | **0.01** |  |  | 0.282/800 SA | 8.0 | **0.0002** |
|  |  | 0.564/400 SA | 1.8 | 0.98 |  |  | 0.564/400 SA | 13.5 | **0.0002** |
|  |  | 0.564/800 SA | 6.0 | **0.004** |  |  | 0.564/800 SA | 14.0 | **0.0002** |
|  |  | 0.282/400 CA | 2.5 | 0.81 |  |  | 0.282/400 CA | 6.7 | **0.0009** |
|  |  | 0.282/800 CA | 4.9 | **0.04** |  |  | 0.282/800 CA | 9.4 | **0.0002** |
|  |  | 0.564/400 CA | 8.0 | **0.0001** |  |  | 0.564/400 CA | 14.0 | **0.0002** |
|  |  | 0.564/800 CA | 16.3 | **0.0001** |  |  | 0.564/800 CA | - | - |
|  | 0.282/400 SA | 0.282/400 CA | 4.5 | 0.09 |  | 0.282/400 SA | 0.282/400 CA | 1.7 | 0.98 |
|  |  | 0.282/800 CA | 12.0 | **0.0001** |  |  | 0.282/800 CA | 4.4 | 0.10 |
|  |  | 0.564/400 CA | 15.0 | **0.0001** |  |  | 0.564/400 CA | 9.0 | **0.0002** |
|  |  | 0.564/800 CA | 23.3 | **0.0001** |  |  | 0.564/800 CA | - | - |
|  | 0.282/800 SA | 0.282/400 CA | 3.0 | 0.60 |  | 0.282/800 SA | 0.282/400 CA | 1.3 | 1.00 |
|  |  | 0.282/800 CA | 10.5 | **0.0001** |  |  | 0.282/800 CA | 1.4 | 1.00 |
|  |  | 0.564/400 CA | 13.6 | **0.0001** |  |  | 0.564/400 CA | 6.0 | **0.004** |
|  |  | 0.564/800 CA | 21.8 | **0.0001** |  |  | 0.564/800 CA | - | - |
|  | 0.564/400 SA | 0.282/400 CA | 4.4 | 0.11 |  | 0.564/400 SA | 0.282/400 CA | 6.8 | **0.0007** |
|  |  | 0.282/800 CA | 3.1 | 0.56 |  |  | 0.282/800 CA | 4.2 | 0.14 |
|  |  | 0.564/400 CA | 6.2 | **0.003** |  |  | 0.564/400 CA | 0.5 | 1.00 |
|  |  | 0.564/800 CA | 14.4 | **0.0001** |  |  | 0.564/800 CA | - | - |
|  | 0.564/800 SA | 0.282/400 CA | 8.6 | **0.0001** |  | 0.564/800 SA | 0.282/400 CA | 7.3 | **0.0003** |
|  |  | 0.282/800 CA | 1.1 | 1.00 |  |  | 0.282/800 CA | 4.6 | 0.06 |
|  |  | 0.564/400 CA | 2.0 | 0.96 |  |  | 0.564/400 CA | 0.0 | 1.00 |
|  |  | 0.564/800 CA | 10.2 | **0.0001** |  |  | 0.564/800 CA | - | - |
